# Global prevalence of urolithiasis: a meta-analysis accounting for methodological heterogeneity

**DOI:** 10.3389/fruro.2025.1705953

**Published:** 2025-12-12

**Authors:** Víctor Juan Vera-Ponce, Nataly Mayely Sanchez-Tamay, Jhosmer Ballena-Caicedo, Fiorella E. Zuzunaga-Montoya, Carmen Inés Gutierrez De Carrillo, Rossmery Leonor Poemape Mestanza

**Affiliations:** 1 Facultad de Medicina (FAMED), Universidad Nacional Toribio Rodríguez de Mendoza de Amazonas (UNTRM), Chachapoyas, Peru; 2 Departamento de Medicina Interna, Hospital Regional Virgen de Fátima, Chachapoyas, Peru

**Keywords:** kidney calculi, nephrolithiasis, prevalence, systematic review, meta-analysis

## Abstract

**Introduction:**

Urolithiasis, also known as renal lithiasis or nephrolithiasis, is an increasingly relevant urological pathology worldwide.

**Objective:**

To estimate the global prevalence of urolithiasis through a systematic review (SR) with meta-analysis, and to systematically investigate methodological sources of heterogeneity in reported prevalence, including differences according to diagnostic methods, sex, and geographical regions.

**Methodology:**

A SR followed PRISMA guidelines adapted for prevalence studies, searching SCOPUS, Web of Science, PubMed, and EMBASE. Observational studies were included in reporting the frequency of urolithiasis diagnosed by ultrasound, tomography, or self-report. A meta-analysis of proportions was performed using a random-effects model with double arcsine transformation. Subgroup analyses by diagnostic method, sex, sampling strategy, and geographical region were conducted. Additionally, meta-regression was conducted to analyze the influence of publication year on prevalence.

**Results:**

In the combined analysis of 22 studies encompassing 1, 276, 826 participants, the estimated global prevalence of urolithiasis was 10.85% (95% CI: 8.76–13.14%). Considerable heterogeneity was observed (I² = 100%). Subgroup analyses revealed that diagnostic methods substantially influenced estimates: ultrasound 8.71% (95% CI: 5.74–12.23%), computed tomography 7.83% (95% CI: 7.12–8.60%), and self-report 13.28% (95% CI: 9.98–16.98%). Probabilistic sampling yielded 8.59% (95% CI: 6.34–11.14%) versus non-probabilistic 12.24% (95% CI: 9.32–15.50%). Prevalence was higher in males (12.93%) than females (8.91%). Regional variation ranged from 22.3% (Africa) to 8.3% (North America). Meta-regression showed publication year had no significant effect when adjusted for methodological factors (p = 0.1304).

**Conclusions:**

Urolithiasis affects approximately 11% of the global population, constituting a public health problem requiring comprehensive preventive, diagnostic, and therapeutic actions. The substantial heterogeneity is largely explained by methodological factors, particularly diagnostic methods and sampling strategies. This highlights the critical importance of standardizing diagnostic and recruitment criteria to obtain comparable measurements for guiding health policies and future research.

## Introduction

Urolithiasis, also known as renal lithiasis or nephrolithiasis, has become a global health concern due to its increasing prevalence in recent decades. This condition is characterized by the formation of calculi in any part of the urinary system, including kidneys (nephrolithiasis), ureters (ureterolithiasis), bladder (cystolithiasis), and urethra (urethrolithiasis). These calculi can cause acute pain, hematuria, infections, and, in severe cases, irreversible renal damage. Furthermore, its management entails substantial costs for healthcare systems in both developed and developing countries, underscoring the importance of deepening our understanding and prevention strategies (1).

Risk factors contributing to kidney stone formation include diet, inadequate hydration, genetic predisposition, climate, overweight, and metabolic alterations. These factors vary according to geographical region and socioeconomic conditions, which explains why the prevalence of urolithiasis is not homogeneous worldwide (2, 3). Nevertheless, epidemiological trends show a global increase, especially in areas with warm climates and significant changes in dietary habits.

Population studies conducted in different countries have documented that the prevalence of urolithiasis ranges between 5% and 15% in adults, with rates that may even exceed these figures in certain risk groups (4). These epidemiological findings reinforce the importance of generating robust evidence to identify common and specific patterns and associated factors to establish more effective preventive and therapeutic strategies.

Although isolated reviews on the epidemiology of urolithiasis exist (2, 5–7), a critical gap remains: the extent to which reported prevalence variation reflects methodological differences (diagnostic criteria, sampling strategies) versus true epidemiological diversity remains poorly quantified. This lack of methodological standardization undermines valid global comparisons and complicates public health planning. In this context, the main objective of the present study is to estimate the global prevalence of urolithiasis through a systematic review (SR) with meta-analysis and to systematically investigate methodological sources of heterogeneity in reported prevalence. Specifically, we examine how diagnostic methods, sampling strategies, and study quality influence prevalence estimates to provide both a reference estimate and methodological context for its interpretation. The results are expected to provide reliable data on the magnitude of this pathology while characterizing the methodological factors that drive variation in reported estimates across different populations (8).

## Methodology

### Design

The present investigation corresponds to a SR with a meta-analysis of studies that evaluated the prevalence of urolithiasis. The process of searching, selecting, extracting, and synthesizing information was carried out following the guidelines established in the PRISMA (Preferred Reporting Items for Systematic Reviews and Meta-Analyses) guide (9), adapted for SRs of prevalence (10). A completed PRISMA 2020 checklist is provided as **Supplementary Material 1**.

### Search strategy

The bibliographic search was conducted in SCOPUS, Web of Science (including the SciELO catalog), PubMed, and EMBASE databases from database inception through May 14, 2025, following the recommendations of the Cochrane Handbook for Systematic Reviews of Interventions to identify the largest amount of relevant literature available (11). For this purpose, standardized terms and synonyms related to urolithiasis were employed, combined through Boolean operators, to maximize the sensitivity of the search strategy. Search terms included “kidney stones, “ “renal calculi, “ “urolithiasis, “ “nephrolithiasis, “ and “prevalence, “ as well as synonyms or controlled vocabulary (MeSH, Emtree) adapted to each database. The detailed design of the strategy, including keywords, specific descriptors, and applied filters, is presented in **Supplementary Material 2**.

### Selection criteria

For this SR, selection criteria were defined to respond clearly and consistently to the research question, focusing on obtaining valid and comparable estimates of the prevalence of urolithiasis globally. This methodological delimitation sought not only to maximize the relevance of the included studies but also to ensure the homogeneity and reliability of the collected data.

Regarding inclusion criteria, observational studies —mainly cross-sectional and cohort studies — that provided quantitative data on the prevalence of urolithiasis in adult populations (≥18 years) were considered. The diagnosis of this pathology could have been performed through imaging tests (ultrasound, radiography, tomography, etc.) or self-reported, provided there was a clear description of how the diagnosis was obtained. Both probabilistic and non-probabilistic sampling methods were accepted, and no restrictions were imposed on the publication language to encompass the broadest possible evidence.

In contrast, studies that exclusively examined populations with specific characteristics were excluded, so the estimates could not be extrapolated to the general population. When multiple publications used the same database or patient cohort, only the most comprehensive study or the one with the largest sample size was included to avoid duplication of data. Likewise, case reports, letters to the editor, SRs, and bibliometric reviews were not considered, as they did not provide primary data on the frequency of urolithiasis.

### Study selection process

The search strategy described in the previous section was systematically applied to each selected database (SCOPUS, Web of Science, PubMed, and EMBASE). The results obtained were exported and imported into the Rayyan reference management software to facilitate the process of duplicate elimination and management of the identified articles.

Two reviewers carried out the selection process independently and in blind mode. Each evaluated the titles and abstracts to determine potential eligibility and examined the full text in cases they considered relevant. After this stage, the blind in Rayyan was lifted so that both reviewers compared and discussed the articles included or excluded in each phase. When discrepancies arose, the reviewers debated until reaching a consensus on the relevance of including or excluding each study. If mutual agreement was not reached, the final decision fell to a third researcher, who reviewed the applied criteria and the publication’s content to issue a final opinion.

### Data extraction and qualitative analysis

Once the selection phase was concluded, the included articles were recorded in a spreadsheet prepared in Excel 2023 to have a centralized database to facilitate subsequent synthesis and analysis. Detailed information on the methodological characteristics and reported results was extracted from each selected study. Variables collected included title, authors, year of publication, country or region of origin, study design (cross-sectional, cohort, or other), sample size, method of diagnosis of urolithiasis (imaging or self-report), inclusion and exclusion criteria, and the main frequency measure.

Additionally, the observed proportion or rate of urolithiasis, the reported confidence interval or standard error, and the distribution by sex were recorded. For the qualitative synthesis, the methodologies employed and the main results of each publication were reviewed, describing the similarities and discrepancies in the inclusion criteria, target population, and measurement of outcomes.

### Risk of bias assessment

Two researchers independently evaluated the risk of bias in the finally included studies using the tool proposed by Munn et al. for prevalence studies (10). This methodology is considered appropriate for reviewing studies that report prevalence estimates due to its ability to assess in detail the methodological quality and consistency in measuring the frequency of the pathology.

The tool includes ten fundamental criteria, among which stand out the representativeness of the sample about the target population, the suitability of the sampling frame, the presence or absence of a random method for participant selection, the minimization of non-response bias, the use of valid and reliable case definitions, consistency in the form of data collection, and the adequacy of the duration of the prevalence period.

Each criterion was classified as “Low risk, “ “High risk, “ or “Unclear.” One point was assigned for each criterion evaluated as “Low risk, “ thus forming a total score for each study. Based on the final score, studies were categorized into three levels of risk: those with 0 to 3 points were considered high risk, 4 to 6 points medium risk, and 7 to 10 points low risk of bias. When discrepancies occurred in the assessment of criteria, they were resolved by consensus or, if necessary, through the intervention of a third reviewer.

### Statistical analysis

Statistical procedures were performed using the R language, version 4.2.2. All studies reporting quantitative data on cases (r) and sample size (n) about urolithiasis were included in the prevalence meta-analysis. The meta prop function from the meta package was used to synthesize the proportion of affected participants robustly.

Proportions were transformed using the Freeman-Tukey method (double arcsine transformation, sm = “PFT”). At the same time, confidence intervals were calculated by applying the Clopper-Pearson method (method.ci = “CP”), which provides exact intervals for proportions. Given the expectation of heterogeneity among studies—originated by differences in the diagnosis of urolithiasis, demographic characteristics of the population, and other contextual factors—a random effects model was chosen with the DerSimonian and Laird estimator (method.tau = “DL”). The Hartung-Knapp correction (hakn = TRUE) was also applied to adjust standard errors. The I² statistic and Cochran’s Q test were analyzed to evaluate heterogeneity, whose values are automatically generated with the metaprop function. The synthesis of results and their 95% confidence intervals were visually represented through forest plots.

Additionally, meta-regressions were performed to explore the possible influence of continuous variables, such as the year of publication and sample size, on the prevalence of urolithiasis. For this purpose, the weighted least squares method was employed, assigning weights inversely proportional to the variance of each study. A mixed effects model was adjusted using the rma function from the metafor package in R. Finally, “bubble” plots were generated in which the size of each bubble was proportional to the statistical weight of each study, which facilitated the visual interpretation of the relationship between the explored factors and the observed prevalence.

## Results

### Article selection

**Figure 1:**
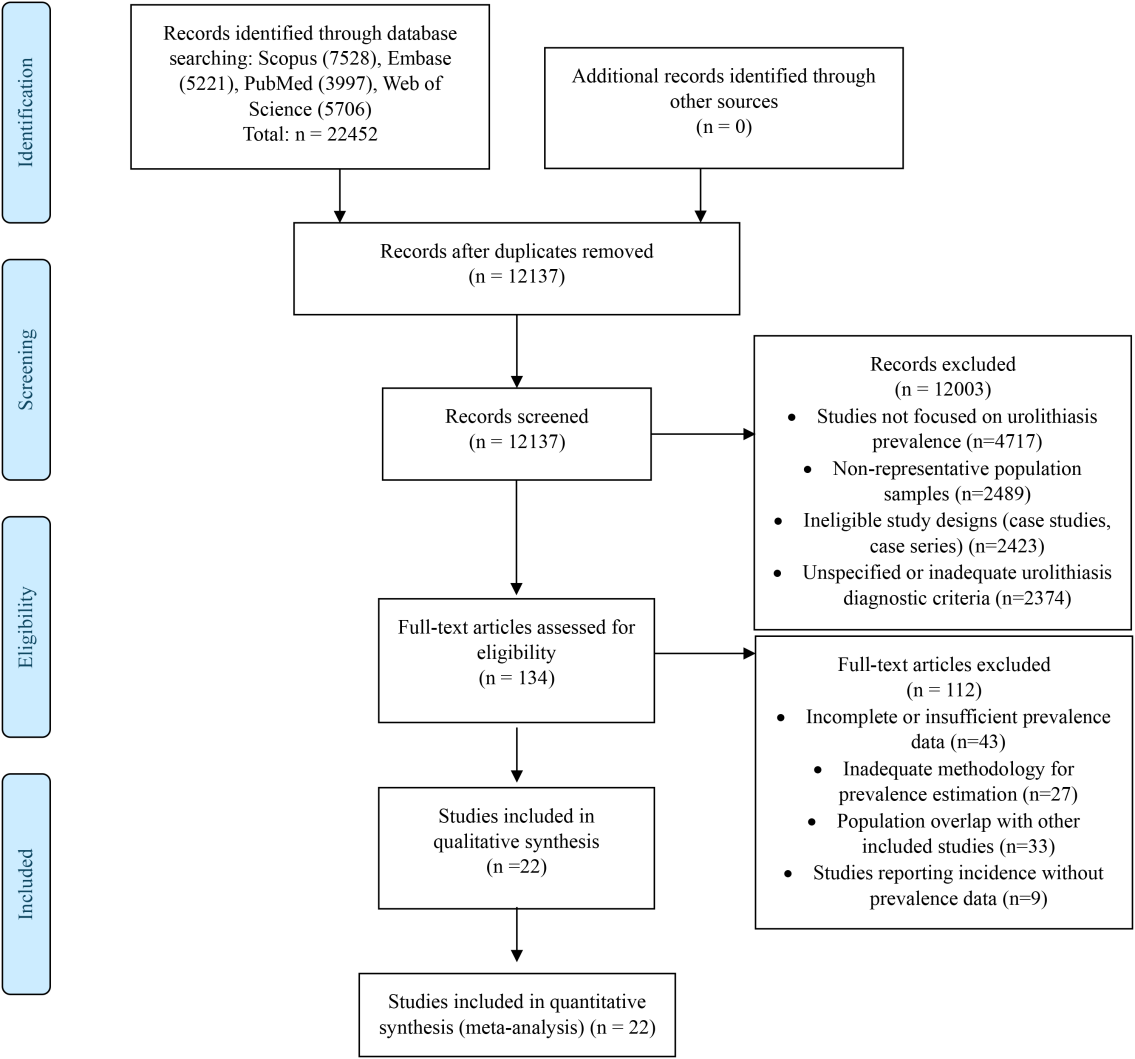
Flowchart of study selection.

Following the PRISMA guidelines, we conducted an exhaustive selection process in the results section. Our initial search in Scopus, Embase, PubMed, and Web of Science databases yielded 22, 452 records, which were reduced to 12, 137 after removing duplicates. After reviewing titles and abstracts, 12, 003 records were excluded. Subsequently, of the 134 full-text articles assessed for eligibility, 112 were excluded. Finally, 22 studies were included in the qualitative synthesis and quantitative meta-analysis (see **Figure 1**) (12–33).

### General characteristics of selected studies

The 22 studies comprising this review were published between 2006 and 2024 (**Table 1**) and include data from countries located in Europe (Greece (12), Italy (14), Serbia (16)), Asia (China (17, 18, 22, 25), Iran (21, 23, 27, 32, 33), Malaysia (28), Saudi Arabia (24, 30), Iraq (19)), Africa (Ethiopia (15)), North America (United States (13, 20, 26, 29)), and the Caribbean (Trinidad and Tobago (31)). Most employed a cross-sectional design (20 studies), while two corresponded to cohorts (13, 14). This geographic and methodological diversity provides a broad view of the frequency of urolithiasis globally.

**Table 1:** Characteristics of the selected studies and risk of bias analysis.

First author	Year	Country	Study type	Sampling	Selection criteria	Sample	Sex (% female)	Age	Diagnosis	Total score*	Risk of bias
Stamatiou	2006	Greece	Cross-sectional	Probabilistic	Residents ≥20 years from urban area of Thebes. Excluded: incomplete questionnaires.	359	54.00%	–	Ultrasound	9	Low
Boyce	2010	United States	Cohort	Non-probabilistic	No specific criteria.	5047	–	56.9	CT	7	Low
Prezioso	2014	Italy	Cohort	Non-probabilistic	Population >17 years with medical care by family medicine specialist registered in database. Minimum two years of recorded medical history.	900994	51.96%	–	Ultrasound	8	Low
Andualem	2014	Ethiopia	Cross-sectional	Non-probabilistic	No specific criteria.	376	–	–	Ultrasound	5	Medium
Fetahu	2016	Serbia	Cross-sectional	Non-probabilistic	No specific criteria.	2506	32.70%	45.9	Self-report	3	High
Zeng	2017	China	Cross-sectional	Probabilistic	General population of China ≥18 years with ≥6 months residence in selected households.	9310	59.27%	51.3	Ultrasound	10	Low
Jiang	2017	China	Cross-sectional	Probabilistic	Adults ≥18 years with local residence >3 years. Excluded: urolithiasis, renal failure, chronic gastric disease, urinary malformations, obstructive diseases or urinary tract infections, hyperparathyroidism.	3350	67.43%	48.97	Ultrasound	9	Low
Tahir	2018	Iraq	Cross-sectional	Non-probabilistic	Excluded: asymptomatic stones, patients undergoing procedures (lithotripsy, stent placement) and presence of microhematuria.	714	49.00%	48.4	Ultrasound	6	Medium
Qin	2021	United States	Cross-sectional	Probabilistic	Adults ≥18 years. Excluded: pregnant women and cases with incomplete data on TyG index or urolithiasis.	20972	51.54%	47.42	Self-report	6	Medium
Khalili	2021	Iran	Cross-sectional	Non-probabilistic	People between 35–70 years from areas with minimal migration rates and different socioeconomic levels. Excluded: incomplete questionnaires.	9932	53.46%	49.94	Self-report	5	Medium
Qiu	2021	China	Cross-sectional	Non-probabilistic	No specific criteria.	11827	44.40%	44.89	Ultrasound	6	Medium
Shahidi	2022	Iran	Cross-sectional	Non-probabilistic	Excluded: illegible or incomplete questionnaires.	556	46.04%	44.69	Self-report	4	Medium
Bokhari	2022	Saudi Arabia	Cross-sectional	Non-probabilistic	Adults ≥18 years residing in the Hail region. Excluded: incomplete questionnaires.	1150	49.10%	26.3	Self-report	4	Medium
Xu	2022	China	Cross-sectional	Non-probabilistic	Adults ≥18 years. Excluded: lack of ultrasound result, renal deformity, kidney transplant, solitary kidney or incomplete clinical evaluation.	98232	43.53%	41.22	Ultrasound	8	Low
Forbes	2022	United States	Cross-sectional	Non-probabilistic	No specific criteria.	114055	61.00%	66	Self-report	6	Medium
Basiri	2023	Iran	Cross-sectional	Probabilistic	No specific criteria.	44186	50.00%	36	Self-report	6	Medium
Perumal	2023	Malaysia	Cross-sectional	Probabilistic	Adults ≥18 years with stay >6 months in respective cities. Excluded: those who denied consent and pregnant women.	1087	55.30%	50.24	Ultrasound	8	Low
Cui	2024	United States	Cross-sectional	Probabilistic	Excluded: <20 years, pregnant women, cases with incomplete information on KSD and CDAI, and extreme CDAI values.	29763	51.20%	–	Self-report	8	Low
Alshubaili	2024	Saudi Arabia	Cross-sectional	Non-probabilistic	Adults ≥18 years residing in Riyadh province.	1043	48.90%	–	Self-report	4	Medium
Persaud	2024	Trinidad and Tobago	Cross-sectional	Non-probabilistic	Adults >18 years residents of Trinidad and Tobago.	1225	53.50%	–	Ultrasound	6	Medium
Babaalizadeh	2024	Iran	Cross-sectional	Probabilistic	People between 35–70 years with residence in the area ≥5 years. Excluded: impediments to completing measurements or questionnaires due to mental or physical disorders.	10133	54.90%	48.63	Self-report	7	Low
Cheraghian	2024	Iran	Cross-sectional	Non-probabilistic	People between 35–70 years residing in Hoveyzeh district, without severe mental disorders and with the ability to respond to questionnaires independently. Excluded: incomplete questionnaires.	10009	59.77%	48.76	Self-report	5	Medium

Also, it is important to highlight that, although our search strategy was broad and included general terms to capture any form of urinary lithiasis, all studies that met the eligibility criteria specifically focused on nephrolithiasis and ureterolithiasis. None of the studies finally included in this review reported specific data on isolated cystolithiasis or urethrolithiasis, consistent with the predominant approach in epidemiological studies of population prevalence. This reflects the current scientific landscape, where renal and ureteral calculi constitute the main focus of research as they represent the vast majority of clinically relevant cases of urolithiasis.

Regarding sample size, studies such as those by Andualem (2014) (15) and Shahidi (2022) (23) analyzed fewer than 1, 000 participants, while others, such as Prezioso (2014) (14) and Xu (2022) (25), exceeded several tens or even hundreds of thousands of subjects. Similarly, the mean age of the evaluated populations frequently ranges between 30 and 70 years, and notable differences in sex distribution are detected. For example, Jiang (2017) (18) included 67.43% of women, in contrast to Basiri (2023) (27), where the female proportion was 50%.

It should be considered that some studies have evaluated specific populations that may not be representative of the general population. Andualem (15) was based on a hospital-based sample of urological patients from a single Ethiopian institution, while Persaud (31), utilized a self-selected online survey methodology that reached 1, 225 participants. Other studies such as those by Khalili (21) and Qin (20) employed broader population-based samples with specific age-related inclusion criteria. These methodological differences in recruitment and population characteristics are reflected in the quality assessment scores and contribute to the heterogeneity observed in prevalence estimates among the included studies.

According to the risk of bias analysis (**Table 1**), nine studies (39.1%) showed low risk (≥7 points), 13 studies (56.5%) presented medium risk (4–6 points), and 1 study (4.3%) was classified as high risk (3 points). The most determinant factors for methodological quality were sample representativeness and selection (criteria 1 and 2), management of response rate (criterion 9), and uniformity in urolithiasis measurement (criteria 6 and 7). It was also observed that studies with probabilistic sampling and imaging diagnosis (ultrasound or CT) tended to record higher scores, suggesting a lower probability of bias.

Finally, the funnel plot for the prevalence of urolithiasis shows an asymmetric distribution of studies, with points scattered in a range of Freeman-Tukey transformed proportions between 0.15 and 0.50. There is a greater concentration of studies in the left portion of the graph (values between 0.15 and 0.30). In contrast, studies in the right region (values >0.35) are more dispersed and present greater variability in their standard errors. This irregular distribution suggests possible publication bias, where studies with higher prevalences of urolithiasis show greater methodological heterogeneity or might be underrepresented in the literature. Studies with lower standard error (upper part of the graph) do not cluster uniformly around the central line, indicating variability not explained by chance and possibly influenced by factors such as population or methodological differences in kidney stone detection (**Supplementary Material 3**).

### Meta-analysis of global and country-specific prevalence of urolithiasis

**Figure 2:**
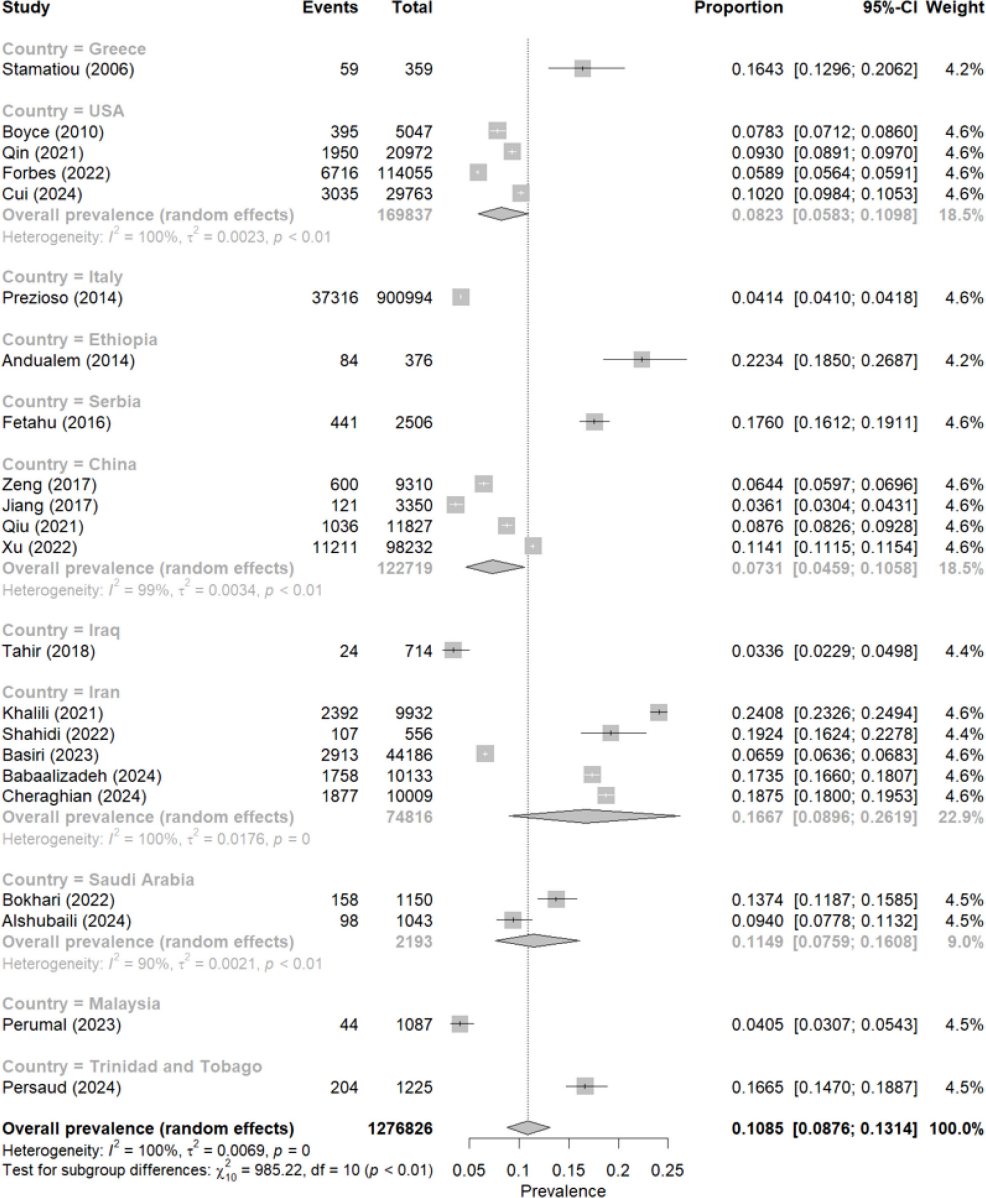
Meta-analysis of the global and country prevalence of urolithiasis.

In the combined analysis of the 22 studies, encompassing 1, 276, 826 participants, the estimated global prevalence of urolithiasis was 10.85% (95% CI: 8.76–13.14%) (see **Figure 2**). In turn, very high heterogeneity was observed (I² = 100%, p < 0.01), indicating substantial differences between studies regarding population, diagnostic methods, and other contextual factors.

When analyzing the prevalence grouped by country, notable variations were evident. For example, in Ethiopia, one of the highest rates was recorded (22.34%) (15), while in China, one of the lowest was reported (3.61%) (18). Other countries with high values include Iran (16.67% in joint estimation of several studies) (21, 23, 27, 32, 33) and Trinidad and Tobago (16.65%) (31), while in Italy, one of the lowest prevalences was observed (4.14%) (14).

### Sensitivity analysis of urolithiasis prevalence

The subgroup evaluation revealed notable differences in the prevalence of urolithiasis according to sex, the diagnostic method employed, and the sampling strategy (see **Table 2**). In the case of sex (14, 16–19, 21, 23–25, 27, 28, 30–33), women presented an estimated prevalence of 8.91% (95% CI: 6.70–11.39%), while in men it reached 12.93% (95% CI: 8.91–17.57%), which suggests that urolithiasis might have a higher frequency in males, although heterogeneity was very high (I² = 100%) in both groups.

**Table 2:** Sensitivity analysis of the prevalence of urolithiasis.

Subgroup	Number of studies	Prevalence %	95% CI	I^2^
Sex				
Female	15	8.91	6.70 – 11.39	100%
Male	15	12.93	8.91 – 17.57	100%
Diagnostic method				
Ultrasound	10	8.71	5.74 – 12.23	100%
CT	1	7.83	7.12 – 8.60	—
Self-report	11	13.28	9.98 – 16.98	100%
Sampling				
Probabilistic	8	8.59	6.34 – 11.14	99%
No probabilistic	14	12.14	9.32 – 15.50	100%
Risk of bias				
Low	9	8.42	5.28 – 12.20	100%

On the other hand, studies that used ultrasound reported, on average, a prevalence of 8.71% (95% CI: 5.74–12.23%) (12, 14, 15, 17–19, 22, 22, 25, 28, 31), while in those based on CT it was lower (7.83%, 95% CI: 7.12–8.60%) (13). In contrast, self-report was associated with a higher value of 13.28% (95% CI: 9.98%–16.98%) (16, 20, 21, 23, 24, 26, 27, 29, 30, 32, 33). These variations could be due to differences in the sensitivity and specificity of each detection method and possible memory biases or underdiagnosis. Likewise, discrepancies were observed between studies with probabilistic sampling (prevalence of 8.59%, 95% CI: 6.34–11.14%) (12, 17, 18, 20, 27–29, 32) and those that adopted non-probabilistic sampling (12.24%, 95% CI: 9.32–15.50%) (13–16, 19, 21–26, 30, 31, 33).

**Figure 3:**
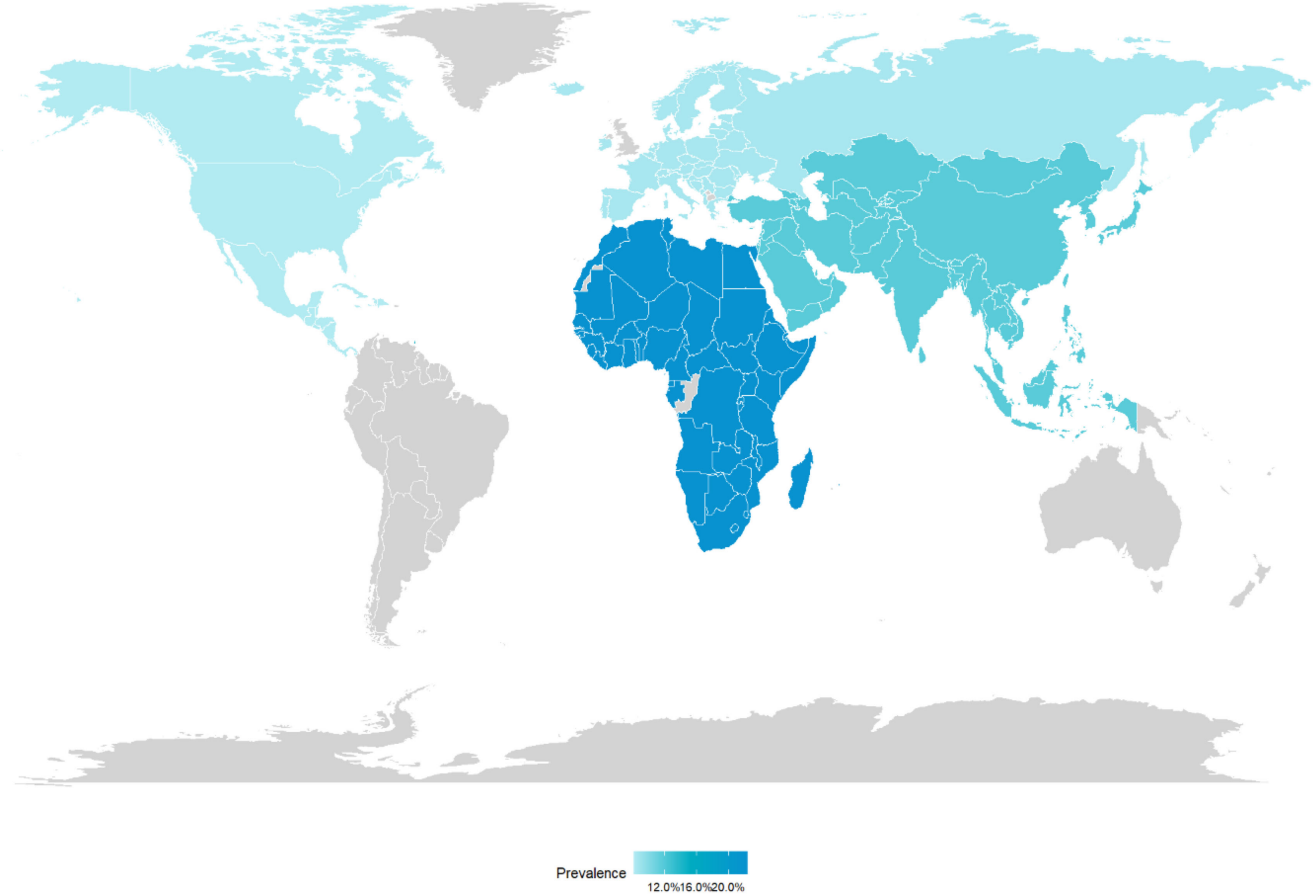
Global distribution of urolithiasis prevalence by region. Values represent pooled prevalence estimates (with 95% confidence intervals) for each geographic region based on available studies. Note: These are unadjusted estimates that reflect both genuine epidemiological differences and methodological heterogeneity across studies within each region.

Regional analysis of urolithiasis prevalence (see **Figure 3**) demonstrated substantial geographical variations. Meta-analyzed prevalence was highest in Africa (22.3%, represented by one study from Ethiopia), followed by the Caribbean (16.7%, one study from Trinidad and Tobago). Asia, with the largest number of included studies (n=13), showed an intermediate prevalence of 11.8%. The lowest prevalence rates were observed in Europe (8.7%, three studies) and North America (8.3%, four studies).

Additionally, when restricting the analysis to studies with low risk of bias (9 studies, n=1, 074, 539), the prevalence was 8.42% (95% CI: 5.28–12.20%), demonstrating robustness of our findings even when limited to methodologically superior studies, though heterogeneity remained substantial (I² = 100%).

### Meta-regression of urolithiasis prevalence by publication year

**Figure 4:**
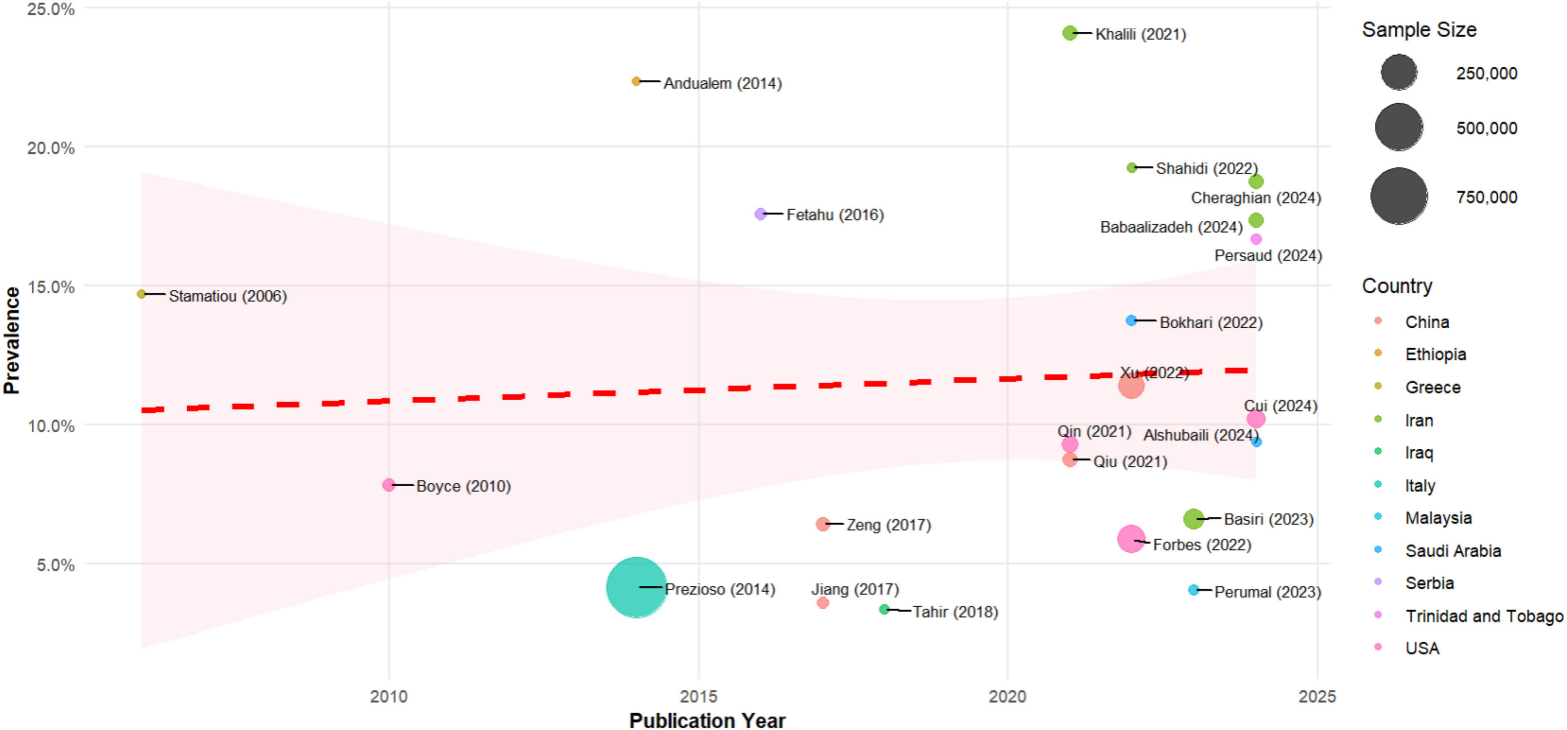
Meta-regression of the prevalence of urolithiasis by year of publication. Red dashed line shows temporal tren with 95% confidence Interval.

Univariate meta-regression revealed a significant temporal trend, with publication year associated with increased prevalence estimates (β = 0.0144, p = 0.0169). However, when adjusted for sex, diagnostic method, and sampling type in the multivariable model, this temporal effect was entirely eliminated (β = 0.0074, p = 0.1304), revealing that the apparent increase in urolithiasis over time was confounded by changes in methodological approaches rather than reflecting genuine epidemiological trends. The multivariable model demonstrated superior fit compared to the year-only model (AIC = -43.5 vs -38.8), indicating that diagnostic method, sex, and sampling approach are more important predictors of prevalence estimates than publication year alone. As illustrated in **Figure 4**, while the regression line shows a modest upward trend across the study period (2006-2024), individual studies display considerable scatter around this trend, with recent studies showing prevalence estimates ranging from approximately 4% to 24%.

## Discussion

### Summary of main findings

This meta-analysis of 22 studies encompassing 1, 276, 826 participants across multiple continents estimated the global prevalence of urolithiasis at 10.85% (95% CI: 8.76–13.14%), indicating that approximately one in ten adults worldwide is affected by this condition. However, substantial heterogeneity was observed across studies (I² = 100%), which is characteristic of prevalence meta-analyses and reflects both genuine epidemiological diversity between populations and methodological variability in how studies are conducted. Our systematic investigation of heterogeneity sources revealed that diagnostic methods and sampling strategies substantially influence reported estimates, with differences of 5.5 percentage points between self-report and CT-based studies, and 3.7 percentage points between non-probabilistic and probabilistic sampling. Understanding these methodological determinants allows appropriate interpretation of the pooled estimate and explains much of the apparent geographic variation observed in the literature. Importantly, sensitivity analysis restricted to studies with low risk of bias yielded a consistent prevalence estimate (8.42%), confirming the robustness of our findings.”Additionally, relevant patterns were identified in subgroups. For example, studies that used imaging diagnosis (ultrasound or computed tomography) tended to report different prevalences than those based solely on self-report, reflecting the influence of sensitivity and specificity of each technique. Similarly, probabilistic sampling was associated with different estimates compared to non-probabilistic methods. These findings highlight the importance of standardizing diagnostic and recruitment criteria to achieve comparable and reliable estimates of urolithiasis worldwide.

Our decision to provide pooled estimates serves a practical public health purpose. Decision-makers often require approximate prevalence figures for resource allocation and intervention planning, even when ideal methodological conditions are not met. We believe our comprehensive sensitivity analyses strengthen rather than weaken our contribution by allowing readers to examine estimates across different diagnostic approaches. Nevertheless, we emphasize that these pooled estimates should be interpreted with considerable caution given the methodological diversity of included studies. Future research should prioritize the development and adoption of standardized diagnostic criteria to enable more homogeneous meta-analyses and reduce the uncertainty inherent in current prevalence estimates.

### Discussion on diagnostic methods and sex differences

The variety of methods employed to diagnose urolithiasis—mainly ultrasound, CT, and self-report—constitutes one factor that explains the heterogeneity observed in prevalence estimates. Ultrasound is often considered a non-invasive and economical alternative, with acceptable sensitivity for moderate to large-sized stones. However, it may underestimate the presence of very small stones or those located in anatomically difficult-to-access regions (4). Meanwhile, due to its high sensitivity and specificity, non-contrast CT is recognized as the reference standard for urolithiasis detection. However, its massive use in population studies is limited by cost and radiation exposure, which justifies the predominance of ultrasound in many contexts. Finally, self-reporting depends on the patient’s memory and previous diagnosis made by a professional; if the patient is unaware of asymptomatic stones, the prevalence measured under this approach may be lower than the actual prevalence (1, 34).

It is important to contextualize the observed differences between diagnostic methods by considering their accuracy rates. Non-contrast CT is regarded as the reference standard, with a sensitivity of 94-99% and specificity of 96-100% for detecting urinary calculi (35), resulting in minimal false-positive (1-4%) and false-negative (1-6%) rates. In contrast, ultrasound presents significantly lower and variable sensitivities, ranging from 40-85% for renal calculi with specificities of 84-98% (35, 36), generating false negative rates of 15-60% that increase considerably for small stones (<5mm) or those located in the mid-ureter. Studies also demonstrate that ultrasound overestimates stone size, particularly for those smaller than 5mm (37). Although methodologically convenient for large-scale epidemiological studies, self-reporting presents inherent limitations due to underdiagnosis of asymptomatic cases and memory biases, which partially explains the higher prevalence observed in studies based on this method.

Our finding that CT-based studies reported a lower prevalence (7.83%) than ultrasound-based studies (8.71%), despite CT’s superior diagnostic sensitivity (94-99% vs. 40-85%), deserves careful consideration, as it appears counterintuitive and highlights fundamental challenges in urolithiasis epidemiology. Examination of the included studies reveals that this paradox likely stems from systematic differences in population selection rather than diagnostic performance per se. The CT-based studies in our meta-analysis (13, 22) both evaluated truly asymptomatic populations undergoing routine screening examinations—colonoscopy screening in Boyce’s study (13) and general health screening in Qiu’s cohort (22). These populations had no urological symptoms and no clinical indication for stone evaluation. In contrast, several ultrasound-based studies included populations with urological symptoms, clinical risk factors (obesity, metabolic syndrome, family history), or were conducted in settings where imaging was clinically indicated (12, 14, 15, 17–19, 25, 28, 31). This fundamental difference in population selection—asymptomatic screening versus clinically indicated evaluation—creates systematic bias that overwhelms the technical advantages of CT.

This paradox illuminates the distinction between radiological prevalence and clinical prevalence. CT’s superior sensitivity detects very small stones (1-3mm) that may never become symptomatic—incidental findings that may pass spontaneously or remain asymptomatic indefinitely. In contrast, ultrasound studies, particularly in symptomatic populations, tend to preferentially detect clinically significant stones. Additionally, ultrasound is often employed in populations with a higher pre-test probability based on symptoms or risk factors, potentially creating selection bias toward a higher prevalence. Furthermore, the reporting thresholds for stone size vary between modalities. This paradox illustrates that diagnostic performance characteristics established in controlled settings do not directly translate to population-level prevalence estimates, where the interplay between diagnostic method, population selection, and disease definitions determines the reported prevalence through mechanisms that extend beyond simple detection capability.

Discrepancies in reported prevalence, depending on the diagnostic test type, highlight the importance of methodological standardization. In research contexts, using homogeneous protocols that include validated diagnostic algorithms could reduce variability between studies and facilitate their comparability (38). Additionally, technique selection is influenced by contextual and economic factors, as the availability of CT or personnel trained for ultrasound in rural settings or those with limited resources is often lower. These logistical and financial considerations must be taken into account when extrapolating prevalences found in a particular study to broader populations or those of a different socioeconomic nature.

The substantial difference in prevalence between probabilistic (8.59%) and non-probabilistic sampling (12.24%) observed in our meta-analysis merits specific discussion, as it demonstrates how sampling methodology profoundly influences prevalence estimates. The nearly 4 percentage point difference we observed suggests that a substantial portion of reported variation in urolithiasis prevalence across studies may reflect sampling methodology rather than true epidemiological differences between populations. This finding has important implications for the interpretation of existing literature and the design of future studies. Studies using non-probabilistic sampling should explicitly acknowledge this limitation and, when possible, compare characteristics of their sample with population demographics to assess representativeness. Future prevalence research should prioritize probabilistic sampling designs whenever feasible, or at a minimum, employ strategies to enhance representativeness, such as quota sampling matched to population demographics or statistical weighting procedures to adjust for selection biases.

On the other hand, the substantial heterogeneity observed in our meta-analysis (I² > 90%) likely stems from multiple factors beyond methodological differences. Regional variations in environmental factors such as climate (particularly temperature and humidity levels), dietary patterns (including salt intake, animal protein consumption, and hydration habits), genetic predisposition, and socioeconomic determinants of healthcare access all potentially contribute to the observed variability in urolithiasis prevalence (2, 3). As Sorokin et al. (4) noted, these epidemiological differences explain why the prevalence of urolithiasis is not homogeneous worldwide. Similarly, Wang et al. (5) demonstrated significant regional variations in mainland China, reinforcing the impact of geographical and lifestyle factors. The influence of dietary habits and inadequate hydration on stone formation and recurrence has been well-documented by Gamage et al. (8), while metabolic factors associated with modern lifestyles have been linked to increasing prevalence trends (1, 3). Future research would benefit from standardized reporting of these contextual factors, enabling more nuanced subgroup analyses and better isolation of key determinants, as recommended by methodological guidelines for epidemiological studies.

Our meta-regression analysis revealed that methodological factors entirely confounded the apparent temporal trend toward increasing prevalence. While univariate analysis showed a significant association between publication year and prevalence (β = 0.0144, p = 0.0169), when adjusted for diagnostic method, sex, and sampling type, this temporal effect was completely eliminated (β = 0.0074, p = 0.1304). This demonstrates that what appears to be increasing disease burden over time actually reflects changes in how studies are conducted rather than true population-level increases. This finding contrasts with epidemiological reports suggesting increasing urolithiasis rates due to modern lifestyle factors such as rising obesity prevalence, increased consumption of processed foods high in sodium and animal protein, and sedentary behaviors (2, 3). Studies have documented associations between urolithiasis and metabolic syndrome components, including obesity and diabetes, which have increased globally over recent decades (20, 22). The influence of dietary habits and inadequate hydration on stone formation has been well-documented (8). However, our analysis reveals that methodological heterogeneity obscures true epidemiological changes, indicating that apparent temporal increases may be artifacts of improved diagnostic capabilities, expanded study populations, or shifts in methodological approaches rather than genuine increases in disease burden. This underscores our central conclusion that methodological heterogeneity is a primary determinant of reported prevalence variation.”

Regarding sex differences, various studies have documented that urolithiasis is historically more common in men, which has been related to hormonal, anatomical, and lifestyle factors (39). For example, testosterone could favor the development of urolithiasis, while estrogen exerts a partial protective effect by modulating the excretion of calcium and other ions (39, 40). However, in recent decades, some analyses have noted a progressive increase in urolithiasis in women, to the point of narrowing the prevalence gap between both sexes (41). This phenomenon could reflect dietary changes, increased obesity incidence, or alterations in physical activity.

Lastly, it is relevant to note that variations related to sex cannot be completely dissociated from socioeconomic and cultural factors since dietary habits, hydration, and access to health services differ among regions and population groups (40). Additionally, selection biases can influence the observed differences. If certain studies included more female participants (or vice versa) or if there was an underrepresentation of males with asymptomatic lithiasis, global estimates would be altered. Facing clinical practice and health policy formulation, it is fundamental to deepen these disparities by sex and refine preventive recommendations, considering both biological characteristics and environmental factors that affect stone formation.

### Clinical and public health implications

In the clinical field, urolithiasis represents one of the most frequent causes of urological emergencies, associated with high healthcare costs and a significant decrease in patients’ quality of life, as well as the loss of working hours. The prevalence identified in this meta-analysis indicates the magnitude of the problem and reinforces the need for healthcare professionals, especially nephrologists and urologists, to recognize the signs and risk factors of the disease in the early stages. Timely identification can prevent complications such as severe obstructions or urinary tract infections, which increase the likelihood of permanent kidney damage.

From a preventive perspective, the estimates provided by this study can guide the development and dissemination of clinical guidelines focused on healthy lifestyle habits. Maintaining adequate water intake, promoting a balanced diet, and encouraging regular physical activity are strategies shown to decrease the risk of stone formation (42, 43). In turn, reducing sodium intake and animal protein, as well as controlling associated diseases such as obesity and diabetes, contribute to the prevention of recurrences in patients with a history of urolithiasis (44, 45).

Regarding public health, the notably high prevalence observed in certain regions suggests that urolithiasis may warrant consideration in national health plans for resource distribution and awareness campaigns, though the methodological heterogeneity in our findings limits the precision of these estimates for specific populations. The potential economic impact of hospital care, surgical procedures, and complications associated with urolithiasis could be substantial for healthcare systems, particularly in countries with limited resources (46), though local epidemiological data would be needed to quantify this burden accurately.

While our findings indicate substantial variation in prevalence across populations, they suggest that designing population screening programs using noninvasive diagnostic techniques like ultrasound might benefit areas with high-risk factors (high environmental temperatures, inadequate dietary habits, etc.), though such strategies must be carefully evaluated considering the availability of trained personnel, investment in diagnostic equipment, and local healthcare priorities. The heterogeneity in our data underscores that adopting these approaches should be balanced with population-specific evidence and awareness about the importance of hydration and periodic surveillance (47).

Finally, coordination between different levels of healthcare is essential to improve the comprehensive approach to urolithiasis. Primary care physicians and specialists must stay updated on international guidelines for preventing and treating the disease, facilitating multidisciplinary management that encompasses dietitians, nutritionists, and endocrinologists, as necessary. Only in this way will it be possible to advance toward a significant reduction in the prevalence and complications associated with urolithiasis, optimize healthcare resources, and improve patients’ quality of life (47).

### The path forward: standardizing urolithiasis epidemiology

The substantial methodological heterogeneity documented in this meta-analysis reveals an important gap in urolithiasis epidemiology: the field lacks consensus standards for prevalence estimation, which limits the comparability and synthesis of findings across studies. We consider that the urological and nephrological research communities could benefit from establishing standardized methodological frameworks for future prevalence studies. Future population-based prevalence studies could prioritize imaging-based diagnosis (ultrasound or computed tomography) over self-report methodologies. When imaging is employed, it would be valuable for studies to explicitly report the diagnostic modality used, technical parameters, criteria for defining urolithiasis, and interpreter qualifications. For resource-limited settings where imaging may not be feasible for large populations, self-report methodologies could be validated through medical record review or imaging confirmation in representative subsamples, with validation results transparently reported.

It would be beneficial for studies to adopt probabilistic sampling designs when feasible, with transparent reporting of sampling methodology, selection procedures, and response rates. When non-probabilistic sampling is necessary due to practical constraints, researchers could clearly acknowledge this limitation and implement strategies to improve representativeness. Minimum sample size requirements could be established based on expected prevalence and desired precision, with *post-hoc* power calculations reported to demonstrate adequacy of achieved sample sizes. It would be useful for all studies to report prevalence stratified by key demographic variables including age, sex, and relevant clinical covariates, as well as geographic information with climate classification and socioeconomic indicators. Confidence intervals, precision measures, and raw data on numerators and denominators could be provided to enable meta-analytic synthesis.

We consider valuable the development of consensus guidelines through coordination among relevant professional societies including the American Urological Association, European Association of Urology, International Society of Nephrology, and epidemiological research consortia. These guidelines could establish minimum methodological standards for prevalence studies while acknowledging resource diversity across settings. International research collaborations using harmonized protocols would generate more comparable data and enable valid geographic comparisons. We recognize that immediate universal adoption of imaging-based diagnosis with probabilistic sampling may not be feasible given resource constraints in many settings. Therefore, we suggest a pragmatic tiered approach: studies could transparently report their methodological approach, allowing future meta-analyses to conduct meaningful comparisons within methodologically homogeneous subgroups.

The methodological heterogeneity documented in our review can serve as a catalyst for change rather than resignation to continued incomparability. Our findings demonstrate that diagnostic methods and sampling strategies profoundly influence reported prevalence—often more than genuine epidemiological differences between populations. This represents both a challenge and an opportunity: by establishing and adhering to standardized methodological practices, the urolithiasis research community can generate the high-quality, comparable epidemiological data necessary for evidence-based public health planning and clinical guideline development.

We hope this meta-analysis serves not only as a reference for current prevalence estimates but as a foundation for improved epidemiological research in urolithiasis. Over time, as methodological standardization increases, the field will generate more precise and comparable prevalence estimates that allow for advances in understanding this condition and improving care for affected patients.

### Strengths and limitations

One of the main strengths of this meta-analysis lies in the breadth of the bibliographic search and the rigorous application of selection methods, following PRISMA guidelines for SRs of prevalence. Likewise, including studies from multiple geographic regions and with various designs allows a more robust approach to the global landscape of urolithiasis by integrating results from populations with different dietary habits, climates, and socioeconomic conditions. Additionally, a standardized protocol was followed for data extraction and risk of bias analysis, which contributes to the internal consistency of the review and strengthens confidence in the obtained estimates.

However, this work has limitations that should be considered when interpreting the findings. First, the high heterogeneity among studies derived from differences in diagnostic methods (self-report, ultrasound, tomography), inclusion criteria, and population characteristics makes it difficult to compare prevalence estimates. Similarly, most cross-sectional studies do not allow for establishing causalities or determining the temporality of kidney stone appearance. Finally, in some studies, non-response rates or selection biases are not described in detail, which could affect the validity of the results and underestimate or overestimate the actual prevalence of urolithiasis.

Finally, another important limitation identified in this study is the apparent contradiction between the theoretical superior sensitivity of CT versus ultrasound for detecting urolithiasis and our meta-analysis results showing higher prevalence estimates in ultrasound-based studies. This discrepancy likely stems from several methodological factors, including the limited number of CT-based studies in our analysis (n=2), potential selection biases in populations undergoing different imaging techniques, and the influence of diverse clinical and epidemiological contexts across studies. This finding highlights the challenges in comparing prevalence estimates across different diagnostic modalities and emphasizes the need for more standardized approaches in future epidemiological research on urolithiasis.

## Conclusions and recommendations

We identified a global prevalence estimate of 10.85% (95% CI: 8.76–13.14%) for urolithiasis, with substantial differences according to diagnostic method, sampling type, and geographic region. The extreme heterogeneity observed (I² > 99%) highlights the complexity of accurately estimating urolithiasis prevalence across diverse populations and methodological approaches, indicating that this estimate should be interpreted as a descriptive overview rather than a precise global figure. The substantial methodological heterogeneity identified in our review underscores the need for coordinated international efforts to establish consensus diagnostic criteria and study protocols for urolithiasis epidemiological research.

Based on our findings, we recommend that future research prioritize the development of standardized diagnostic protocols with clear criteria for urolithiasis identification, as these methodological differences substantially influenced prevalence estimates in our analysis. The adoption of probabilistic sampling strategies would improve the representativeness and comparability of future studies, though when non-probability sampling is unavoidable, researchers should implement methodological safeguards and explicitly acknowledge limitations in generalizability. While our descriptive findings provide insight into the potential burden of urolithiasis globally, longitudinal studies with standardized methodologies would be necessary to establish temporal trends and understand disease progression, which would be essential for developing evidence-based public health policies and clinical management guidelines.

## Data Availability

The data analyzed in this study is subject to the following licenses/restrictions: Data are available upon request to the corresponding author. Requests to access these datasets should be directed to victor.vera@untrm.edu.pe.

## References

[1] Stamatelou K Goldfarb DS . Epidemiology of kidney stones. Healthc Basel Switz. (2023) 11:424. doi: 10.3390/healthcare11030424, PMID: 36766999 PMC9914194

[2] Emami E Heidari-Soureshjani S Oroojeni Mohammadjavad A Sherwin CM . Obesity and the risk of developing kidney stones: A systematic review and meta-analysis. Iran J Kidney Dis. (2023) 1:63–72. doi: 10.52547/ijkd.722, PMID: 37060339

[3] Lu Z Chen Y Tang Z Zhang J Li Z Tang F . Basal metabolic rate and the risk of urolithiasis: a two-sample Mendelian randomization study. World J Urol. (2024) 42:235. doi: 10.1007/s00345-024-04946-x, PMID: 38616238

[4] Sorokin I Mamoulakis C Miyazawa K Rodgers A Talati J Lotan Y . Epidemiology of stone disease across the world. World J Urol. (2017) 35:1301–20. doi: 10.1007/s00345-017-2008-6, PMID: 28213860

[5] Wang W Fan J Huang G Li J Zhu X Tian Y . Prevalence of kidney stones in mainland China: A systematic review. Sci Rep. (2017) 7:41630. doi: 10.1038/srep41630, PMID: 28139722 PMC5282506

[6] Ferraro PM Curhan GC D’Addessi A Gambaro G . Risk of recurrence of idiopathic calcium kidney stones: analysis of data from the literature. J Nephrol. (2017) 30:227–33. doi: 10.1007/s40620-016-0283-8, PMID: 26969574

[7] Piana A Basile G Masih S Bignante G Uleri A Gallioli A . Kidney stones in renal transplant recipients: A systematic review. Actas Urol Esp. (2024) 48:79–104. doi: 10.1016/j.acuroe.2023.08.003, PMID: 37574010

[8] Gamage KN Jamnadass E Sulaiman SK Pietropaolo A Aboumarzouk O Somani BK . The role of fluid intake in the prevention of kidney stone disease: A systematic review over the last two decades. Turk J Urol. (2020) 46:S92–103. doi: 10.5152/tud.2020.20155, PMID: 32525478 PMC7731957

[9] Page MJ McKenzie JE Bossuyt PM Boutron I Hoffmann TC Mulrow CD . The PRISMA 2020 statement: an updated guideline for reporting systematic reviews. BMJ. (2021) 372:n71. doi: 10.1136/bmj.n71, PMID: 33782057 PMC8005924

[10] Munn Z Moola S Lisy K Riitano D Tufanaru C . Methodological guidance for systematic reviews of observational epidemiological studies reporting prevalence and cumulative incidence data. Int J Evid Based Healthc. (2015) 13:147–53. doi: 10.1097/XEB.0000000000000054, PMID: 26317388

[11] Cochrane Handbook for Systematic Reviews of Interventions (2021). Available online at: https://training.cochrane.org/handbook (Accessed March 1, 2025).

[12] Stamatiou KN Karanasiou VI Lacroix RE Kavouras NG Papadimitriou VT Chlopsios C . Prevalence of urolithiasis in rural Thebes, Greece. Rural Remote Health. (2006) 6:610. doi: 10.22605/RRH610, PMID: 17155848

[13] Boyce CJ Pickhardt PJ Lawrence EM Kim DH Bruce RJ . Prevalence of urolithiasis in asymptomatic adults: objective determination using low dose noncontrast computerized tomography. J Urol. (2010) 183:1017–21. doi: 10.1016/j.juro.2009.11.047, PMID: 20092842

[14] Prezioso D Illiano E Piccinocchi G Cricelli C Piccinocchi R Saita A . Urolithiasis in Italy: an epidemiological study. Arch Ital Urol Androl Organo Uff Soc Ital Ecogr Urol E Nefrol. (2014) 86:99–102. doi: 10.4081/aiua.2014.2.99, PMID: 25017588

[15] Andualem D Gidena G . Admission patterns and management of urolithiasis: A hospital based study in tikur anbessa specialized hospital(TASH), Addis Ababa, Ethiopia. East Cent Afr J Surg. (2014) 19:29–34.

[16] Fetahu A Neziri A Tartari F Karamitri G Qeva O Daku A . Epidemiological research and clinical urolithiasis in Presevo Valley. Ukrainian Sci Pract J Urologists Andrologists Nephrologists. (2014) 22–5.

[17] Zeng G Mai Z Xia S Wang Z Zhang K Wang L . Prevalence of kidney stones in China: an ultrasonography based cross-sectional study. BJU Int. (2017) 120:109–16. doi: 10.1111/bju.13828, PMID: 28236332

[18] Jiang YG He LH Luo GT Zhang XD . Prevalence of kidney stones and associated risk factors in the Shunyi District of Beijing, China. Hong Kong Med J Xianggang Yi Xue Za Zhi. (2017) 23:462–9. doi: 10.12809/hkmj164904, PMID: 28416732

[19] Tahir NL Hassan QA Kamber HM . The prevalence of a clinically silent nephrolithiasis in Baghdad population: an initial ultrasound screening study from Iraq. Acta Med Iran. (2019) 57:51–6. doi: 10.18502/acta.v57i1.1753

[20] Qin Z Zhao J Geng J Chang K Liao R Su B . Higher triglyceride–glucose index is associated with increased likelihood of kidney stones. Front Endocrinol. (2021) 12:774567. doi: 10.3389/fendo.2021.774567, PMID: 34912299 PMC8667164

[21] Khalili P Jamali Z Sadeghi T Esmaeili-Nadimi A Mohamadi M Moghadam-Ahmadi A . Risk factors of kidney stone disease: a cross-sectional study in the southeast of Iran. BMC Urol. (2021) 21:141. doi: 10.1186/s12894-021-00905-5, PMID: 34625088 PMC8499392

[22] Qiu F Xu Y Ji X Pu J Zhou J Huang Y . Incidence and correlation of metabolic syndrome and kidney stones in a healthy screening population. Transl Androl Urol. (2021) 10:3646–55. doi: 10.21037/tau-21-689, PMID: 34733660 PMC8511539

[23] Shahidi S Dolatkhah S Mortazavi M Atapour A Aghaaliakbari F Meamar R . An epidemiological survey on kidney stones and related risk factors in the Iranian community. Acta Med Iran. (2022) 60:307–12. doi: 10.18502/acta.v60i5.9558

[24] Bokhari AA Aldarwish HA Alsanea SA Al-Tufaif MA Alghaslan SA Alghassab AA . Prevalence and risk factors of urolithiasis among the population of Hail, Saudi Arabia. Cureus. (2022) 14:e26983. doi: 10.7759/cureus.26983, PMID: 35989769 PMC9381884

[25] Xu J-Z Li C Xia Q-D Lu J-L Wan Z-C Hu L . Sex disparities and the risk of urolithiasis: a large cross-sectional study. Ann Med. (2022) 54:1627–35. doi: 10.1080/07853890.2022.2085882, PMID: 35675329 PMC9196832

[26] Forbes CM Nimmagadda N Kavoussi NL Xu Y Bejan CA Miller NL . Kidney stone prevalence based on self-report and electronic health records: insight into the prevalence of active medical care for kidney stones. Urology. (2023) 173:55–60. doi: 10.1016/j.urology.2022.11.009, PMID: 36435346 PMC10038847

[27] Basiri A Kashi AH Salehi Omran H Borumandnia N Golshan S Narouie B . National lifetime prevalence and demographic factors of urolithiasis in Iran. Urol J. (2023) 20:102–8. doi: 10.22037/uj.v20i.7576, PMID: 36744405

[28] Perumal KR Chua RHB Teh GC Lei CCM . Prevalence of urolithiasis in Sarawak and associated risk factors: An ultrasonagraphy-based cross-sectional study. BJUI Compass. (2023) 4:74–80. doi: 10.1002/bco2.152, PMID: 36569506 PMC9766857

[29] Cui J Xiao Y Wang J Yin S Huang K Wang J . Association between the composite dietary antioxidant index and prevalence of kidney stone disease in adults: A nationally representative cross-sectional study. J Funct Foods. (2024) 117:106253. doi: 10.1016/j.jff.2024.106253

[30] Alshubaili AM Alotaibi AF Alsaleh KA Almogarri AI Alanizi AA Alsaif SS . The prevalence of nephrolithiasis and associated risk factors among the population of the Riyadh Province, Saudi Arabia. Cureus. (2024) 16:e55870. doi: 10.7759/cureus.55870, PMID: 38595876 PMC11002709

[31] Persaud SA Jankie S Andrews R Varachhia S Morris M . High self-reported prevalence of kidney stones in Trinidad and Tobago: results of a cross-sectional online survey. Cureus. (2024) 16:e57651. doi: 10.7759/cureus.57651, PMID: 38707028 PMC11070117

[32] Babaalizadeh B Taghinezhad A Bijani M Dehghan A . Relationship between body mass index and blood lipids with kidney stone disease: the Fasa adults cohort study. Jundishapur J Chronic Dis Care. (2024) 13:e141674. doi: 10.5812/jjcdc-141674

[33] Cheraghian B Meysam A Hashemi SJ Hosseini SA Malehi AS Khazaeli D . Kidney stones and dietary intake in adults: a population-based study in southwest Iran. BMC Public Health. (2024) 24:955. doi: 10.1186/s12889-024-18393-1, PMID: 38575950 PMC10993538

[34] Eisenberg JM . Imaging Tests to Check for Kidney Stones in the Emergency Department. In: Comparative Effectiveness Review Summary Guides for Consumers AHRQ Comparative Effectiveness Reviews. Agency for Healthcare Research and Quality (US, Rockville (MD (2005). Available online at: http://www.ncbi.nlm.nih.gov/books/NBK379839/. Center for Clinical Decisions and Communications Science (Accessed March 1, 2025)., PMID:

[35] Vijayakumar M Ganpule A Singh A Sabnis R Desai M . Review of techniques for ultrasonic determination of kidney stone size. Res Rep Urol. (2018) 10:57–61. doi: 10.2147/RRU.S128039, PMID: 30128307 PMC6089602

[36] Brisbane W Bailey MR Sorensen MD . An overview of kidney stone imaging techniques. Nat Rev Urol. (2016) 13:654–62. doi: 10.1038/nrurol.2016.154, PMID: 27578040 PMC5443345

[37] Sternberg KM Eisner B Larson T Hernandez N Han J Pais VM . Ultrasonography significantly overestimates stone size when compared to low-dose, noncontrast computed tomography. Urology. (2016) 95:67–71. doi: 10.1016/j.urology.2016.06.002, PMID: 27289025

[38] Spivacow FR Del Valle EE Lores E Rey PG . Kidney stones: Composition, frequency and relation to metabolic diagnosis. Med (Mex). (2016) 76:343–8. 27959841

[39] Monico CG Milliner DS . Genetic determinants of urolithiasis. Nat Rev Nephrol. (2011) 8:151–62. doi: 10.1038/nrneph.2011.211, PMID: 22183508 PMC3901084

[40] Ferraro PM Cunha TDS Curhan GC . Sex differences and the risk of kidney stones. Semin Nephrol. (2022) 42:230–5. doi: 10.1016/j.semnephrol.2022.04.012, PMID: 35718369

[41] Cicerello E Mangano MS Cova G Ciaccia M . Changing in gender prevalence of nephrolithiasis. Urologia. (2021) 88:90–3. doi: 10.1177/0391560320966206, PMID: 33084513

[42] Finger M Finger E Bellucci A Malieckal DA . Medical management for the prevention of kidney stones. Postgrad Med J. (2023) 99:112–8. doi: 10.1136/postgradmedj-2021-140971, PMID: 37222048

[43] Zayed S Goldfarb DS Joshi S . Popular diets and kidney stones. Adv Kidney Dis Health. (2023) 30:529–36. doi: 10.1053/j.akdh.2023.10.002, PMID: 38453270

[44] Ferraro PM Bargagli M Trinchieri A Gambaro G . Risk of kidney stones: influence of dietary factors, dietary patterns, and vegetarian-vegan diets. Nutrients. (2020) 12:779. doi: 10.3390/nu12030779, PMID: 32183500 PMC7146511

[45] Ferraro PM Bargagli M . Dietetic and lifestyle recommendations for stone formers. Arch Esp Urol. (2021) 74:112–22., PMID: 33459627

[46] Garbens A Pearle MS . Causes and prevention of kidney stones: separating myth from fact. BJU Int. (2021) 128:661–6. doi: 10.1111/bju.15532, PMID: 34192414

[47] Assadi F Moghtaderi M . Preventive kidney stones: continue medical education. Int J Prev Med. (2017) 8:67. doi: 10.4103/ijpvm.IJPVM_17_17, PMID: 28966756 PMC5609393

