# Supplementary material for: Global prevalence of urolithiasis: a meta-analysis accounting for methodological heterogeneity
Source: Front Urol. 2025 Dec 12;5:1705953. doi: 10.3389/fruro.2025.1705953 (PMC12740863; doi:10.3389/fruro.2025.1705953)
Supplement: Supplementary file 1 [file DataSheet1.pdf]

Supplementary Material 1. PRISMA 2020 Checklist

| Section/Topic | Item # | Checklist Item | Location Where Item Is Reported |
|---|---|---|---|
| **TITLE** | | | |
| Title | 1 | Identify the report as a systematic review. | Title, page 1 |
| **ABSTRACT** | | | |
| Abstract | 2 | See the PRISMA 2020 for Abstracts checklist. | Abstract, page 1-2 |
| **INTRODUCTION** | | | |
| Rationale | 3 | Describe the rationale for the review in the context of existing knowledge. | Introduction, paragraphs 1-3, page 2-3 |
| Objectives | 4 | Provide an explicit statement of the objective(s) or question(s) the review addresses. | Introduction, final paragraph, page 3 |
| **METHODS** | | | |
| Eligibility criteria | 5 | Specify the inclusion and exclusion criteria for the review and how studies were grouped for the syntheses. | Methods - Selection Criteria, page 3-4 |
| Information sources | 6 | Specify all databases, registers, websites, organisations, reference lists and other sources searched or consulted to identify studies. Specify the date when each source was last searched or consulted. | Methods - Search Strategy, page 3 |
| Search strategy | 7 | Present the full search strategies for all databases, registers and websites, including any filters and limits used. | Methods - Search Strategy, page 3; Supplementary Material 2 (full strategy) |
| Selection process | 8 | Specify the methods used to decide whether a study met the inclusion criteria of the review, including how many reviewers screened each record and each report retrieved, whether they worked independently, and if applicable, details of automation tools used in the process. | Methods - Study Selection Process, page 4 |
| Data collection process | 9 | Specify the methods used to collect data from reports, including how many reviewers collected data from each report, whether they worked independently, any processes for obtaining or confirming data from study investigators, and if applicable, details of automation tools used in the process. | Methods - Data Extraction and Qualitative Analysis, page 4 |
| Data items | 10a | List and define all outcomes for which data were sought. Specify whether all results that were compatible with each outcome domain in each study were sought (e.g. for all measures, time points, analyses), and if not, the methods used to decide which results to collect. | Methods - Data Extraction and Qualitative Analysis, page 4 |
| | 10b | List and define all other variables for which data were sought (e.g. participant and intervention characteristics, funding sources). Describe any assumptions made about any missing or unclear information. | Methods - Data Extraction and Qualitative Analysis, page 4 |
| Study risk of bias assessment | 11 | Specify the methods used to assess risk of bias in the included studies, including details of the tool(s) used, how many reviewers assessed each study and whether they worked independently, and if applicable, details of automation tools used in the process. | Methods - Risk of Bias Assessment, page 4-5 |
| Effect measures | 12 | Specify for each outcome the effect measure(s) (e.g. risk ratio, mean difference) used in the synthesis or presentation of results. | Methods - Statistical Analysis, page 5 |
| Synthesis methods | 13a | Describe the processes used to decide which | Methods - Statistical |

| | | | |
|---|---|---|---|
| | | studies were eligible for each synthesis (e.g. tabulating the study intervention characteristics and comparing against the planned groups for each synthesis (item #5)). | Analysis, page 5 |
| | 13b | Describe any methods required to prepare the data for presentation or synthesis, such as handling of missing summary statistics, or data conversions. | Methods - Statistical Analysis, page 5 |
| | 13c | Describe any methods used to tabulate or visually display results of individual studies and syntheses. | Methods - Statistical Analysis, page 5; Results - Figures 2-4 |
| | 13d | Describe any methods used to synthesize results and provide a rationale for the choice(s). If meta-analysis was performed, describe the model(s), method(s) to identify the presence and extent of statistical heterogeneity, and software package(s) used. | Methods - Statistical Analysis, page 5 (random-effects model, DerSimonian-Laird, $I^2$ statistic, R version 4.2.2) |
| | 13e | Describe any methods used to explore possible causes of heterogeneity among study results (e.g. subgroup analysis, meta-regression). | Methods - Statistical Analysis, page 5 (subgroup analyses, meta-regression) |
| | 13f | Describe any sensitivity analyses conducted to assess robustness of the synthesized results. | Results - Sensitivity Analysis, page 8-9 (low risk of bias subgroup) |
| Reporting bias assessment | 14 | Describe any methods used to assess risk of bias due to missing results in a synthesis (arising from reporting biases). | Results - General Characteristics, page 7 (funnel plot); Supplementary Material 3 |
| Certainty assessment | 15 | Describe any methods used to assess certainty (or confidence) in the body of evidence for an outcome. | Not applicable (prevalence study, not intervention study with GRADE assessment) |
| **RESULTS** | | | |
| Study selection | 16a | Describe the results of the search and selection process, from the number of records identified in the search to the number of studies included in the review, ideally using a flow diagram. | Results - Article Selection, page 6; Figure 1 (PRISMA flowchart) |
| | 16b | Cite studies that might appear to meet the inclusion criteria, but which were excluded, and explain why they were excluded. | Figure 1 reports excluded studies; full exclusion list available upon request |
| Study characteristics | 17 | Cite each included study and present its characteristics. | Results - General Characteristics, page 6-7; Table 1 |
| Risk of bias in studies | 18 | Present assessments of risk of bias for each included study. | Results - General Characteristics, page 7; Table 1 (risk of bias scores) |
| Results of individual studies | 19 | For all outcomes, present, for each study: (a) summary statistics for each group (where appropriate) and (b) an effect estimate and its precision (e.g. confidence/credible interval), ideally using structured tables or plots. | Results, page 7-9; Figure 2 (forest plot with individual study estimates and CI); Table 2 (subgroup results) |
| Results of syntheses | 20a | For each synthesis, briefly summarise the characteristics and risk of bias among contributing studies. | Results - Meta-analysis, page 7-8 |
| | 20b | Present results of all statistical syntheses conducted. If meta-analysis was done, present for each the summary estimate and its precision (e.g. confidence/credible interval) and measures of statistical heterogeneity. If | Results - Meta-analysis, page 7-8 (pooled prevalence 10.85%, 95% CI: 8.76–13.14%, $I^2$=100%); Table 2 |

| | | | |
|---|---|---|---|
| | | comparing groups, describe the direction of the effect. | (subgroup results) |
| | 20c | Present results of all investigations of possible causes of heterogeneity among study results. | Results - Sensitivity Analysis, page 8-9; Table 2 (diagnostic method, sampling, sex, region, risk of bias subgroups) |
| | 20d | Present results of all sensitivity analyses conducted to assess the robustness of the synthesized results. | Results - Sensitivity Analysis, page 8-9 (low risk of bias: 8.42%) |
| Reporting biases | 21 | Present assessments of risk of bias due to missing results (arising from reporting biases) for each synthesis assessed. | Results - General Characteristics, page 7; Supplementary Material 3 (funnel plot) |
| Certainty of evidence | 22 | Present assessments of certainty (or confidence) in the body of evidence for each outcome assessed. | Not applicable (prevalence study) |
| **DISCUSSION** | | | |
| Discussion | 23a | Provide a general interpretation of the results in the context of other evidence. | Discussion, pages 10-14 |
| | 23b | Discuss any limitations of the evidence included in the review. | Discussion - Strengths and Limitations, page 15 |
| | 23c | Discuss any limitations of the review processes used. | Discussion - Strengths and Limitations, page 15 |
| | 23d | Discuss implications of the results for practice, policy, and future research. | Discussion - Clinical and Public Health Implications, pages 13-14; The Path Forward, page 14-15 |
| **OTHER INFORMATION** | | | |
| Registration and protocol | 24a | Provide registration information for the review, including register name and registration number, or state that the review was not registered. | Not registered (retrospective systematic review) |
| | 24b | Indicate where the review protocol can be accessed, or state that a protocol was not prepared. | Protocol not publicly registered |
| | 24c | Describe and explain any amendments to information provided at registration or in the protocol. | Not applicable |
| Support | 25 | Describe sources of financial or non-financial support for the review, and the role of the funders or sponsors in the review. | Funding section, page 17 (funded by Vicerectorado de Investigación de la UNTRM) |
| Competing interests | 26 | Declare any competing interests of review authors. | Conflict of Interest section, page 17 (no conflicts declared) |
| Availability of data, code and other materials | 27 | Report which of the following are publicly available and where they can be found: template data collection forms; data extracted from included studies; data used for all analyses; analytic code; any other materials used in the review. | Data Availability section, page 17 (data available upon request to corresponding author) |

Supplementary material 2. Search strategy

| Search strategy in PUBMED | |
|---|---|
| #1 | "Kidney Calculi"[Mesh] OR "Nephrolithiasis"[Mesh] OR "Urolithiasis"[Mesh] OR "kidney stone"[tiab] OR "kidney stones"[tiab] OR nephrolithiasis[tiab] OR "renal calculi"[tiab] OR "renal calculus"[tiab] OR urolithiasis[tiab] OR "cálculos renales"[tiab] OR "calculos renales"[tiab] OR "litiasis renal"[tiab] OR "piedras en el riñón"[tiab] OR "piedras en el rinon"[tiab] |
| #2 | prevalence[tiab] OR "prevalence"[Mesh] OR epidemiology[tiab] OR "epidemiology"[Mesh] OR frequency[tiab] OR "frecuencia"[tiab] OR "prevalencia"[tiab]) |
| #3 | #1 AND #2 |
| Search strategy in SCOPUS | |
| #1 | TITLE-ABS-KEY ( "Kidney Calculi" OR "Nephrolithiasis" OR "Urolithiasis" OR "kidney stone" OR "kidney stones" OR nephrolithiasis OR "renal calculi" OR "renal calculus" OR urolithiasis OR "cálculos renales" OR "calculos renales" OR "litiasis renal" OR "piedras en el riñón" OR "piedras en el rinon" ) |
| #2 | TITLE-ABS-KEY ( prevalence OR epidemiology OR frequency OR frecuencia OR prevalencia) |
| #3 | #1 AND #2 |
| Search strategy in Web of Science | |
| #1 | TS=( "Kidney Calculi" OR "Nephrolithiasis" OR "Urolithiasis" OR "kidney stone" OR "kidney stones" OR nephrolithiasis OR "renal calculi" OR "renal calculus" OR urolithiasis OR "cálculos renales" OR "calculos renales" OR "litiasis renal" OR "piedras en el riñón" OR "piedras en el rinon" ) |
| #2 | TS=(prevalence OR epidemiology OR frequency OR frecuencia OR prevalencia) |
| #3 | #1 AND #2 |
| Search strategy in EMBASE | |
| #1 | ('kidney calculi':ti,ab,kw OR 'nephrolithiasis':ti,ab,kw OR 'urolithiasis':ti,ab,kw OR 'kidney stone':ti,ab,kw OR 'kidney stones':ti,ab,kw OR nephrolithiasis:ti,ab,kw OR 'renal calculi':ti,ab,kw OR 'renal calculus':ti,ab,kw OR urolithiasis:ti,ab,kw OR 'cálculos renales':ti,ab,kw OR 'calculos renales':ti,ab,kw OR 'litiasis renal':ti,ab,kw OR 'piedras en el riñón':ti,ab,kw OR 'piedras en el rinon':ti,ab,kw) |
| #2 | (prevalence:ti,ab,kw OR epidemiology:ti,ab,kw OR frequency:ti,ab,kw OR frecuencia:ti,ab,kw OR prevalencia:ti,ab,kw) |
| #4 | #1 AND #2 AND #3 |

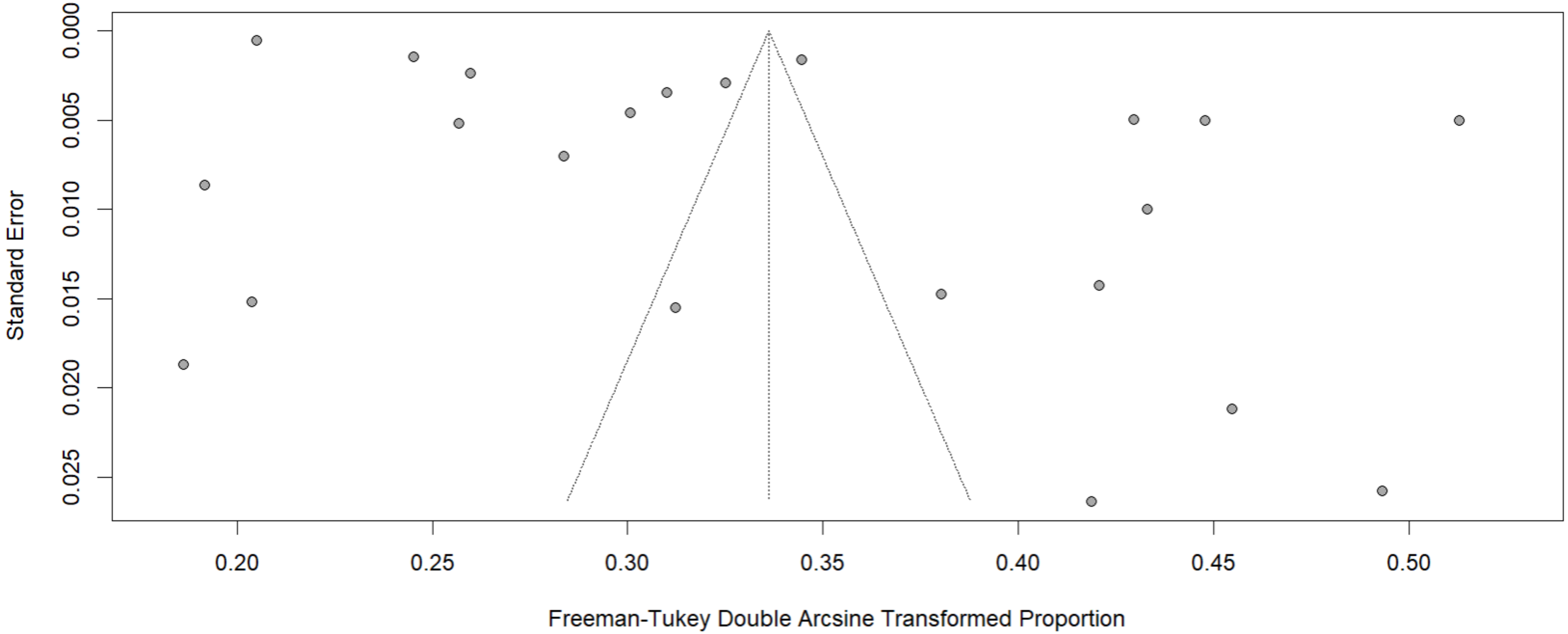

Supplementary Material 3. Funnel Plot of the prevalence of urolithiasis
